# ASC modulates HIF-1α stability and induces cell mobility in OSCC

**DOI:** 10.1038/s41419-020-02927-7

**Published:** 2020-09-03

**Authors:** Chi-Sheng Wu, Ian Yi-Feng Chang, Jui-lung Hung, Wei-Chao Liao, Yi-Ru Lai, Kai-Ping Chang, Hsin-Pai Li, Yu-Sun Chang

**Affiliations:** 1 Molecular Medicine Research Center, Chang Gung University, No.259, Wenhua 1st Rd., Guishan Dist, 333 Taoyuan City, Taiwan Republic of China; 2 Department of Otolaryngology-Head & Neck Surgery, Chang Gung Memorial Hospital at Linkou, 33305 Gueishan, Taoyuan Taiwan; 3 Department of Nephrology, Chang Gung Memorial Hospital, Lin-kou Medical Center, Taoyuan, Taiwan; 4 Department of Microbiology and Immunology, Molecular Medicine Research Center, Chang Gung University, No.259, Wenhua 1st Rd., Guishan Dist., Lin-Kou, 333 Taoyuan, Taiwan Republic of China; 5 College of Medicine, Chang Gung University, Taoyuan, Taiwan; 6 Division of Hematology-Oncology, Department of Internal Medicine, Chang Gung Memorial Hospital, Chang Gung University, No.5, Fuxing St., Guishan Dist, 333 Taoyuan City, Lin-Kou, Taiwan Republic of China

**Keywords:** Oral cancer, Cancer microenvironment

## Abstract

High-level expression of ASC (Apoptosis-associated speck-like protein containing a CARD) leads to lymph node metastasis in OSCC, but the underlying mechanism remains unclear. Here, we show that HIF-1α participates in ASC-induced metastasis. We identified 195 cell-motion-associated genes that were highly activated in ASC-overexpressed SAS_ASC cells; of them, 14 representative genes were found to be overexpressed in OSCC tissues in our previously reported RNA-seq dataset, OSCC-Taiwan. Nine of the 14 genes were also upregulated in OSCC-TCGA samples. Among the nine genes, *RRAS2*, *PDGFA*, and *VEGFA*, were correlated with poor overall survival of patients in OSCC-TCGA dataset. We further demonstrated that the promoters of these 14 ASC-induced genes contained binding motifs for the transcription-regulating factor, HIF-1α. We observed that ASC interacted with and stabilized HIF-1α in both the cytoplasm and the nucleus under normoxia. Molecules involved in the HIF-1α pathway, such as VHL and PHD2, showed no difference in their gene and protein levels in the presence or absence of ASC, but the expression of HIF-1α-OH, and the ubiquitination of HIF-1α were both decreased in SAS_ASC cells versus SAS_con cells. The migration and invasion activities of SAS_ASC cells were reduced when cells were treated with the HIF-1α synthesis inhibitor, digoxin. Taken together, our results demonstrate that the novel ASC-HIF-1α regulatory pathway contributes to lymph node metastasis in OSCC, potentially suggesting a new treatment strategy for OSCC.

## Introduction

Oral squamous cell carcinoma (OSCC), which occurs in the oral cavity, is the most common cause of head and neck cancer^1^. OSCC can be found on the tongue, buccal region, lips, gingiva, and hard palate. It is an aggressive cancer that leads to death; in Taiwan, it is the fifth most common cancer, and has a high mortality rate. The major treatment for OSCC is surgical removal of the tumor mass combined with radiotherapy or chemotherapy for the primary tumor. Metastases of OSCC, however, are difficult to treat with traditional surgery. Tumor cell invasion is a first step for metastasis, and a number of invasion-associated biomarkers and signaling pathway components have been identified as hallmarks for oral cancer progression, including extracellular matrix (ECM) molecules, matrix metalloproteases, cell adhesion molecules, growth factors, and inflammation-associated cytokines^2,3^.

Inflammasome activation is an inflammatory response that promotes the maturation of the cytokines, IL-1β and IL-18^4^. Inflammasome signaling is induced by an interaction between an intracellular sensor (a NOD-like receptor) and Apoptosis-associated speck-like protein containing a CARD (ASC); this forms the caspase-1-activating complex, which in turn activates IL-1β and IL-18. Although the role of inflammasomes in cancer is controversial, they are reportedly involved in the progression of many cancers, including lung, breast, gastric, liver, nasopharyngeal cancer^5^, and OSCC^6,7^. A single prior study showed that overexpression of ASC, which is a key adaptor protein of inflammasome signaling, promoted tumor formation and cancer metastasis^8^. We also demonstrated that ASC is involved in the cervical lymph node metastasis of OSCC^6^. We additionally reported that the promoter region of *ASC* was hypomethylated in OSCC in association with overexpression of ASC at both gene and protein levels^6^, in contrast to other reports showing that ASC was downregulated in several cancers through hypermethylation of the promoter CpG sites^9–11^. Overexpression of ASC can lead to lymph node metastasis and is correlated with poorer overall survival (OS), disease free survival (DFS), and disease specific survival (DSS) of OSCC patients^6^, but the mechanism underlying these associations remains unclear.

Hypoxia is a crucial microenvironmental condition for tumor pathophysiology, including tumor metastasis, and HIF-1α is a key molecule that is highly expressed under hypoxia. In the HIF-1α biogenesis pathway, HIF-1α protein is hydroxylated at Pro_402_ and Pro_564_ by prolyl hydroxylase domain-containing protein 2 (PHD2). HIF-1α-OH is recognized by von Hippel–Lindau (VHL) protein and degraded by ubiquitination within 5–10 min of this recognition^12,13^. When not degraded, HIF-1α interacts with HIF-1β to form a heterodimer, translocating into the nucleus and leading to transcription of downstream genes^14^. During cancer progression, numerous tumor-associated genes are upregulated by HIF-1α through its binding to HIF response elements (HREs) under hypoxia^15,16^. HIF-1α is considered to be a potential prognostic marker of many cancers, including OSCC^17^, and HIF-1α overexpression has been correlated with tumor stage, lymph node metastasis, and poor survival in OSCC^18^. However, the mechanism through which ASC acts on HIF-1α to promote metastasis in OSCC remains unknown.

To examine the mechanism by which ASC induces lymph node metastasis in OSCC, we used RNA sequencing (RNA-seq) to analyze gene expression in cells with/without overexpressing ASC. We found that the majority of the differentially expressed genes contained HREs in their promoters, suggesting that HIF-1α plays an important role in ASC-induced metastasis. We observed that the HIF-1α protein was stabilized by ASC under normoxia, which was similar with cells under hypoxia. We found that ASC and HIF-1α colocalized in both the cytoplasm and the nucleus, as assessed by immunofluorescence and co-immunoprecipitation assays. The genes that appeared to be regulated by HIF-1α in ASC-overexpressing cells were significantly elevated in RNA-seq data obtained from tumor tissues annotated in the OSCC-Taiwan and OSCC-TCGA databases. The three targeted genes *RRAS2*, *PDGFA*, and *VEGFA* were correlated with the OS of OSCC-TCGA patients. Collectively, our novel results reveal that ASC induces lymph node metastasis in OSCC via the stabilization of HIF-1α.

## Results

### HIF-1α regulates cell-motion-associated genes in SAS_ASC cells and OSCC patients

ASC is known to play important biological roles in inflammasome activation and tumorigenesis. In a previous study, we demonstrated that ASC is overexpressed in OSCC, as determined using qRT-PCR data from 20 normal/tumor paired clinical samples and immunohistochemistry scoring data from 111 OSCC patients^6^. Here, we further confirmed that the gene expression level of ASC was elevated in RNA-seq results obtained from 39 normal/tumor paired samples of the Taiwan-OSCC database^19^ and 308 OSCC versus 30 normal clinical samples in the TCGA database. Indeed, ASC gene expression was 1.74-fold and 2.09-fold higher in the OSCC samples of the OSCC-Taiwan and TCGA datasets, respectively (Supplementary Fig. 1, *p* < 0.0001 in both datasets).

**Fig. 1:**
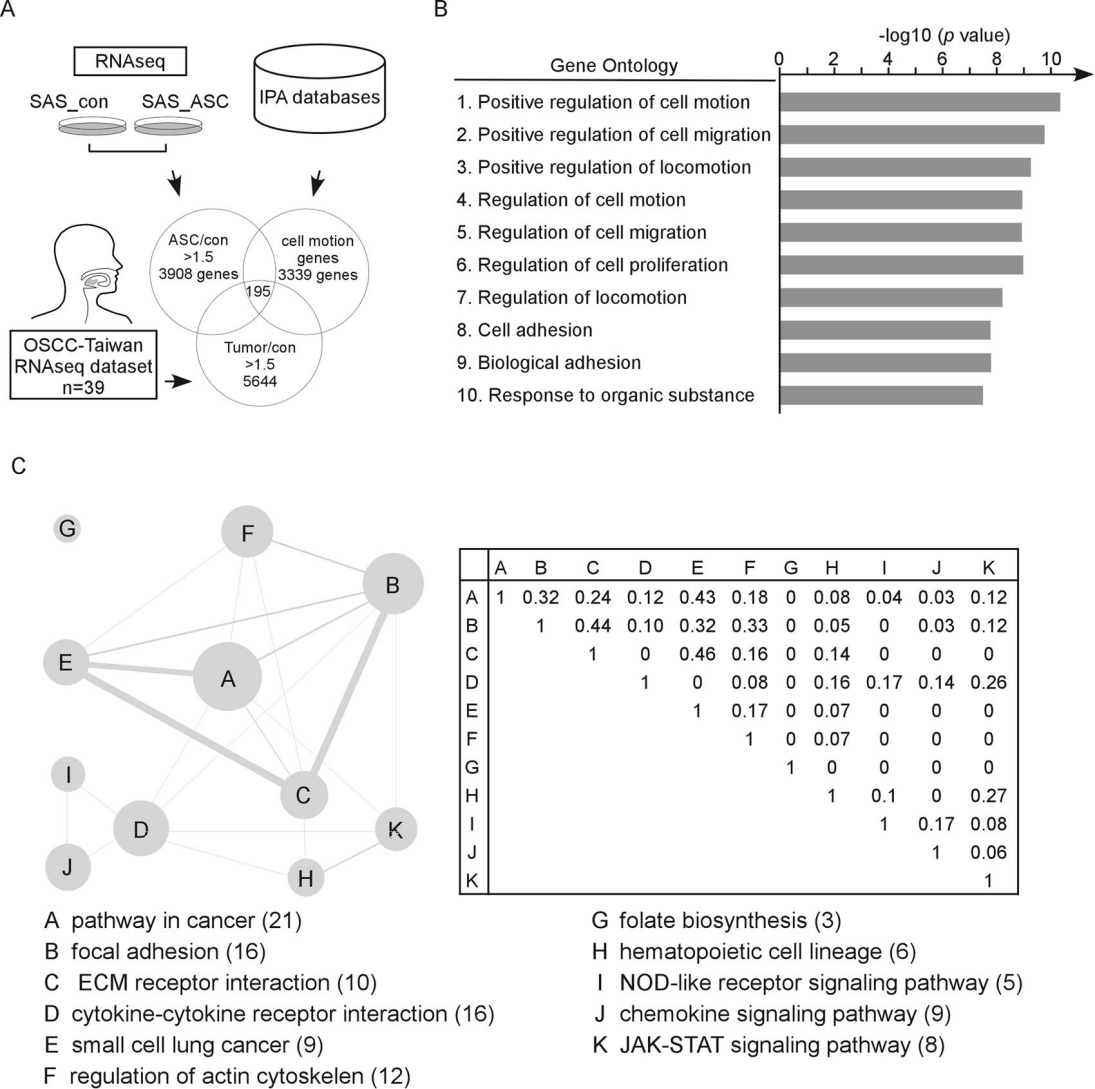
Identification of cell-motion-associated genes upregulated in SAS_ASC cells and OSCC patients. **a** Schematic representation of the cell-motion-associated genes selected from RNA-seq data of SAS_con/SAS_ASC cells, OSCC-Taiwan samples, and databases of cell-motion-associated genes. **b** Gene Ontology analysis of 195 identified cell-motion-associated genes. **c** Pathway analysis of 195 cell-motion-associated genes. The gene numbers are represented by the size of each gray circle and marked in the pathway legends (left). The correlation factors within pathways are indicated by the thickness of each gray stick (right).

In order to investigate genes that might be involved in the ability of ASC to promote OSCC metastasis, we integrated three datasets to identify the potential genes that involved in the OSCC cell mobility induced by overexpression of ASC. Initially, we used RNA-seq to assess the mRNA levels of genes in SAS_con and SAS_ASC cells. This analysis identified 3908 genes that were upregulated by 1.5-fold or more in SAS_ASC cells compared to SAS_con cells (Fig. 1a). Next, we collected 3339 cell migration/invasion-associated genes from the Ingenuity Pathway Analysis (IPA) database. Finally, we examined the OSCC-Taiwan dataset^19^, and found 5644 genes that were shown with 1.5-fold or more elevation in tumor versus normal tissue samples. The intercept of the three datasets revealed that 195 genes with metastatic ability in OSCC (Fig. 1a), which were further subjected to the Gene Ontology (GO) and pathway analyses. Indeed, GO analysis suggested that these genes were mainly involved in cell motion and cell proliferation (https://david.ncifcrf.gov/) (Fig. 1b, Supplementary Table 1, ranked by *p* value). It is worthy to note that the category shown as “response to organic substance” also covers the genes involved in activity of cells, such as gene expression, enzyme production, and cell movement. Similarly, the majority of 195 genes played pivotal roles in cancer pathway regulation, focal adhesion, ECM interaction, actin cytoskeleton regulation, and JAK-STAT signaling, all of which have been correlated with tumorigenesis.

Further analysis revealed that within this dataset, the ECM receptor interaction pathway was highly cross-related with the cancer and focal adhesion pathways (Jaccard coefficients = 0.46 and 0.44, respectively)^20^. Some of the ASC-induced genes were known to upregulate genes involved in cytokine-receptor interactions, chemokine signaling pathways, and the NOD-like receptor signaling pathway, suggesting that ASC can induce inflammation-associated pathways. We also found that folate biosynthesis and hematopoietic cell lineage pathways were upregulated in SAS_ASC cells (Fig. 1c and Supplementary Table 2).

### Cell-motion-associated genes were regulated by HIF-1α protein

**Fig. 2:**
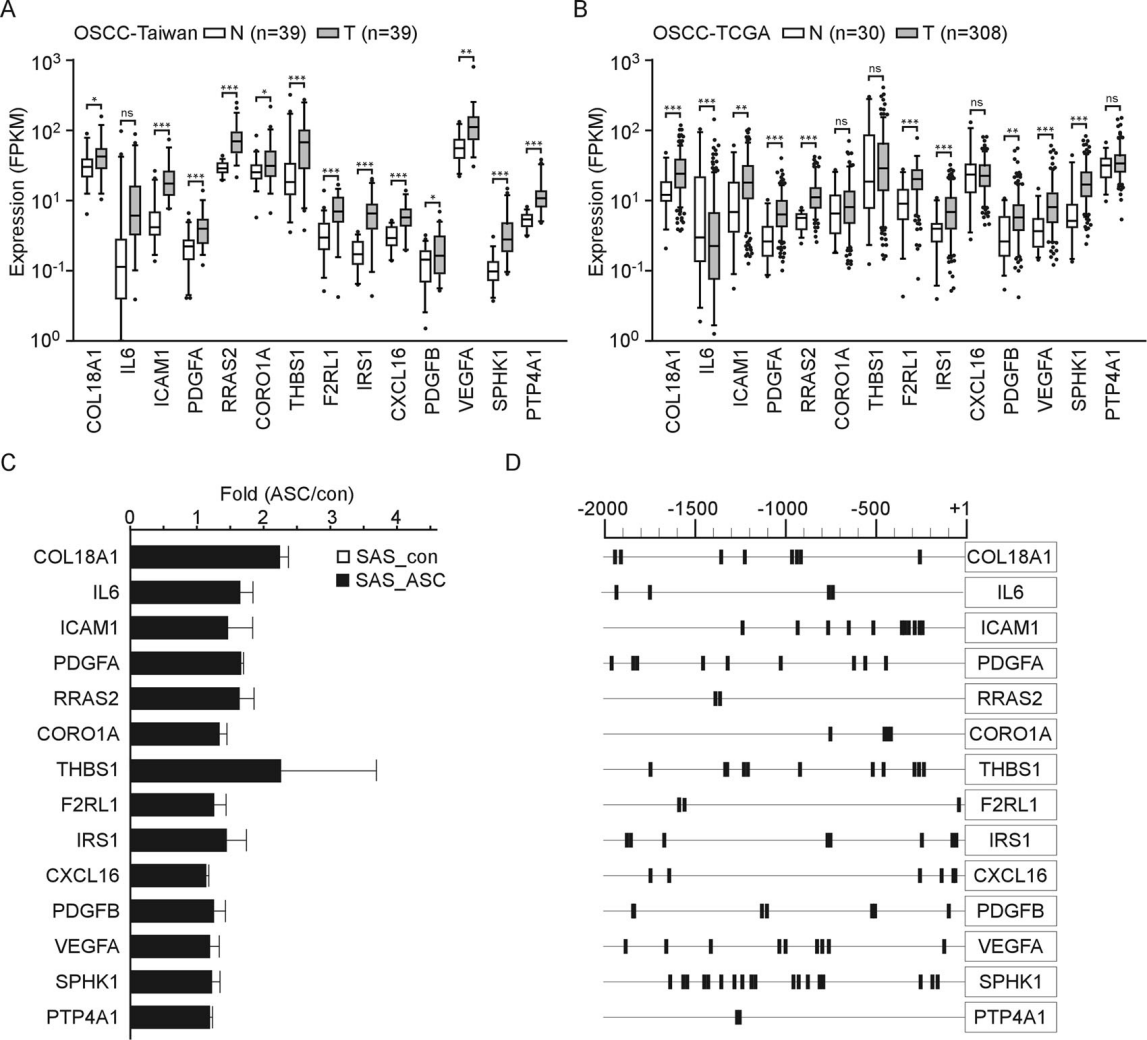
Expression levels of ASC-induced cell-motion genes in OSCC-Taiwan, OSCC-TCGA, and SAS_ASC cells. **a** Expression levels of 14 representative cell-motion-associated genes found to be upregulated in OSCC-Taiwan were assessed in SAS_ASC cells using qRT-PCR. **b** The levels of the 14 representative cell-motion-associated genes in the OSCC-TCGA datasets. **c** Expression level of the 14 ASC-regulated cell-motion-associated genes in SAS_ASC cells. **d** Prediction of potential HRE sites from −2000 to +1 in the promoter regions of the representative cell-motion genes. Black sticks indicate potential HRE sites with the consensus ACGT sequence.

From among the 195 genes that were upregulated in SAS_ASC cells, we selected for validation 14 that were also included in the relevant GO categories (Fig. 1b). All 14 genes were upregulated in the tumor tissues versus the paired adjacent normal regions of 39 patients of OSCC-Taiwan (Fig. 2a). Similar results were obtained when we analyzed the RNA-seq results of 308 OSCC samples compared with 30 healthy individuals, as annotated in the TCGA dataset. In addition to *CORO1A*, *THBS1, PTP4A1, IL6*, and *CXCL16*, the other nine genes were also upregulated in tumor tissues in TCGA (Fig. 2b). Of the 14 genes, the expression levels of nine were positively correlated with the expression of ASC (*r* > 0.4, *p* < 0.05) in the Taiwan-OSCC samples (Supplementary Table 3). Within them, higher-level expression of *RRAS2* and *PDGFA* was correlated with poor survival in the TCGA dataset (*p* = 0.01 and 0.03, respectively). *VEGFA* expression was also associated with poor overall survival in TCGA (*p* = 0.035) (Supplementary Fig. 2), which was consistent with the findings of our previous study^21^. We used qRT-PCR to further validate the gene expression levels of the selected genes, and found that all 14 were upregulated in SAS_ASC cells (Fig. 2c). Because these genes were correlated with cell mobility, we suggested they may potentially be able to involve in the lymph node metastasis in our previous study. Notably, the PDGFA, CORO1A, ICAM1, and THBS1 proteins were overexpressed in SAS_ASC cells. In SAS_ASC cells were treated with siRNA specific to ASC, these four proteins were downregulated (Supplementary Fig. 3A, B). In our previously reported proteomic data, we also found upregulation of ICAM1, RRAS2, CORO1A, and THBS1 in OSCC (Supplementary Fig. 3C)^22^. These results suggest that the expression of select cell-motion-associated genes may be regulated by ASC. However, the underlying mechanism remained unclear.

We hypothesized that these cell-motion-associated genes could be regulated by transcription factors or pathways known to be associated with ASC, such as AP-1 and STAT3^23^. To identify potential upstream regulators of these cell-motion-associated genes, we subjected the RNA-seq data generated from SAS_con and SAS_ASC cells to IPA Upstream Regulator Analysis. We found 508 upstream regulators of the genes found to be differentially expressed between the two cell lines; of them, 66 belonged to transcription factors. Among the transcription factors, 27 were activated in SAS_ASC cells, and most of their differentially expressed target genes were upregulated (z-score > 2, *p* < 0.01). Importantly, HIF-1α was the major activated transcription factor that appeared to be responsible for regulating the downstream genes in SAS_ASC cells (Supplementary Table 4, *p* = 3.63 × 10^−34^). We analyzed the HRE sites from nt −2000 to +1 in the promoter regions of the 14 selected ASC-regulated genes according to the conserved HRE core sequence, ACGT^15,24,25^ (http://jaspar.genereg.net/). Indeed, we identified multiple HRE sites located in the promoter regions of these genes (Fig. 2d). Based on these results, we speculate that ASC may regulate these genes through HIF-1α.

### The HIF-1α protein is stabilized in SAS_ASC cells

**Fig. 3:**
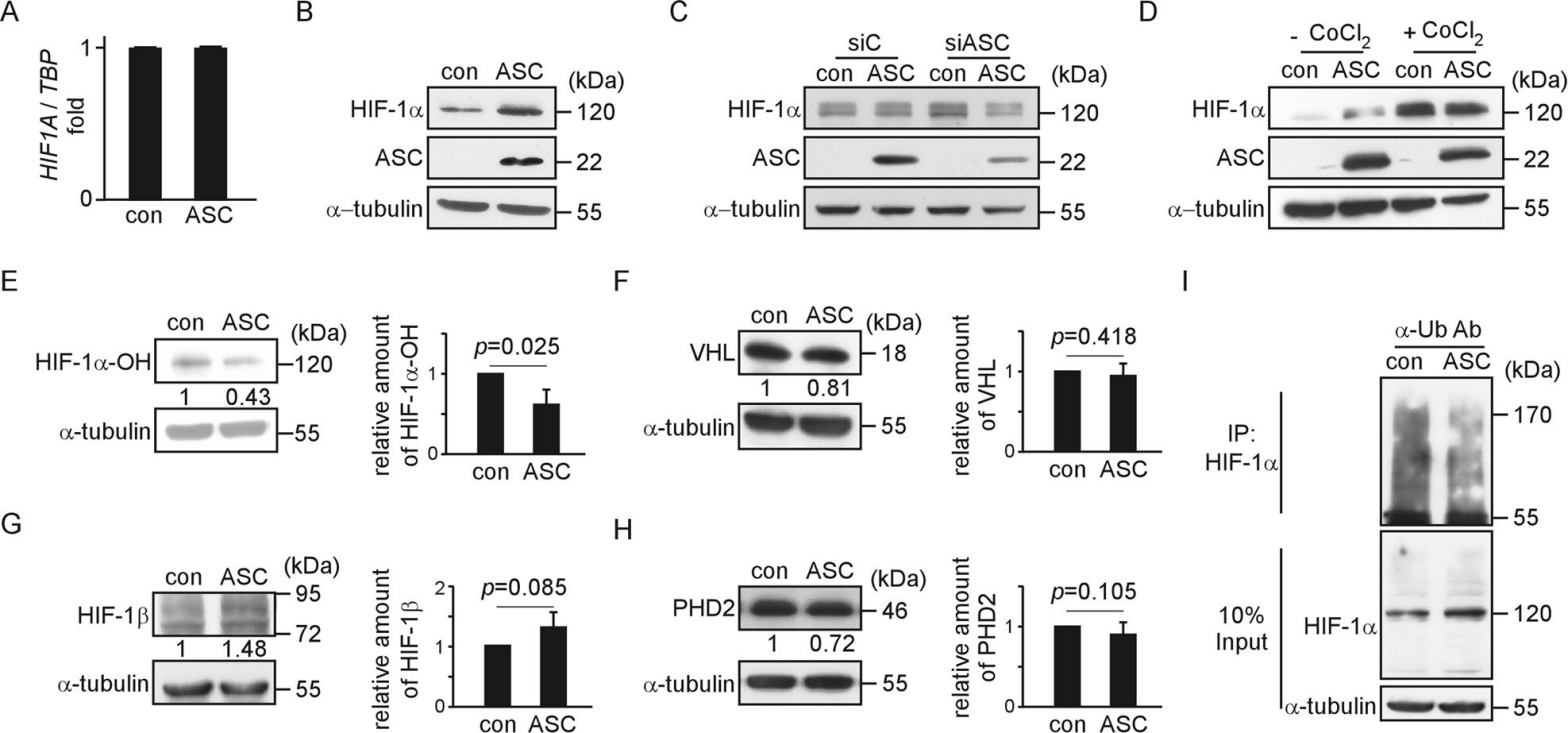
HIF-1α is stabilized in the SAS_ASC cell line. **a** The gene expression levels of *HIF1A* are similar between SAS_con and SAS_ASC cells. The *y*-axis shows the *HIF1A* expression level, as measured by qRT-PCR and normalized to the *TBP* expression level. **b** The HIF-1α protein level is upregulated in SAS_ASC cells, as assessed by western blot analysis. **c** The HIF-1α protein level is downregulated in SAS_ASC cells treated with siRNA specific to ASC. **d** The HIF-1α level in SAS_ASC cells under normoxia is similar to that in SAS_ASC cells treated with CoCl_2_. **e** The representative western blot of HIF-1α−ΟH. The HIF-1α−ΟΗ protein level is 0.62 ± 0.19 fold decreased in SAS_ASC cells from three independent experiments (*p* = 0.025). **f** The representative western blot of VHL. The VHL protein level shows no change between SAS_con and SAS_ASC cell lines from three independent experiments (*p* = 0.418). **g** The representative western blot of HIF-1β. The HIF-1β protein level shows no change between SAS_con and SAS_ASC cell lines from three independent experiments (*p* = 0.085). **h** The representative western blot of PHD2. The PHD2 protein level shows no change between SAS_con and SAS_ASC cell lines from three independent experiments (*p* = 0.105). **i** The ubiquitination of HIF-1α is decreased in SAS_ASC cells. Data were presented as mean ± SD.

HIF-1α is a transcription factor that is induced under hypoxia. In the tumor microenvironment, the central region of the tumor mass is expected to be hypoxic. Here, we hypothesized that, in addition to being induced under low oxygen tension, HIF-1α may also be induced by ASC under normoxic conditions. We examined *HIF1A* expression using qRT-PCR, and found that the *HIF1A* gene expression level did not differ between SAS_con and SAS_ASC cells under normoxia (Fig. 3a). In HIF-1α biogenesis pathway, HIF-1α is synthesized and quickly undergoes ubiquitin-mediated degradation within 10 min under normoxia; in contrast, HIF-1α protein activity is prolonged under hypoxia^12^. Unexpectedly, we found that the HIF-1α protein level was increased in SAS_ASC relative to SAS_con cell lines under normoxia (Fig. 3b, Supplementary Fig. 4A). After ASC was depleted using specific siRNA, HIF-1α was decreased in SAS_ASC cells but not in SAS_con cells (Fig. 3c, Supplementary Fig. 4B). These results indicate that ASC can stabilize the HIF-1α protein. To examine whether the consequence of this increase of HIF-1α in SAS_ASC cells was similar to that seen under hypoxia, we mimicked hypoxia using CoCl_2_, which inhibits the activities of PHD2 that prevents the degradation of HIF-1α by hydroxylating it at Pro_402_ and Pro_564_. Our results revealed that the HIF-1α protein level was increased in both SAS_con and SAS_ASC cells treated with CoCl_2_ (Fig. 3d, Supplementary Fig. 4C). Thus, the ASC-mediated stabilization of HIF-1α appears similar to that seen in cells subjected to CoCl_2_ treatment under normoxia. Together, our results demonstrate that ASC may regulate the stability of HIF-1α to upregulate the levels of HIF-1α-regulated genes in SAS_ASC cells under normoxia.

### ASC interacts with HIF-1α in SAS_ASC cells

The stability of HIF-1α is known to be altered by the amount of HIF-1α-OH that is a transitional mediator molecule in the HIF-1α biogenesis pathway. In that, the amount of HIF-1α is reverse correlated with HIF-1α-OH. We therefore speculated that the stabilization of HIF-1α in SAS_ASC cells could be monitored by the amount of HIF-1α-OH. Indeed, as shown in Fig. 3e and Supplementary Fig. 4D, the amount of HIF-1α-OH was decreased by 0.62 ± 0.19 fold in SAS_ASC cells compared to SAS_con cells (*p* = 0.025). Other important components involved in the HIF-1α biogenesis pathway show no significant changes: VHL protein with 0.96 ± 0.14-fold (*p* = 0.418, Fig. 3f and Supplementary Fig. 4e), HIF-1β/ARNT with 1.32 ± 0.24-fold (*p* = 0.085, Fig. 3g and Supplementary Fig. 4F), and PHD2 with 0.97 ± 0.16-fold (*p* = 0.641, Fig. 3h and Supplementary Fig. 4G) in SAS_ASC cells compared to SAS_con cells. Notably, the gene expression levels of *VHL*, *PHD2*, and *HIF1B* did not significantly differ between the two cell lines (Supplementary Fig. 4H). Since HIF-1α-OH is degraded by the ubiquitin-proteasome pathway, we examined the HIF-1α proteasome degradation status in SAS_con and SAS_ASC cells. As shown in Fig. 3i and Supplementary Fig. 4I, HIF-1α showed more ubiquitination in SAS_con cells than in SAS_ASC cells. Above all, out data indicate that ASC can prevent the hydroxylation and degradation of HIF-1α. Based on these results, we propose that, under normoxia, more HIF-1α is available to interact with HIF-1β and translocate into the nucleus to activate downstream cell-motion-associated genes in SAS_ASC cells compared to the corresponding control cells.

### ASC and HIF-1α colocalize in OSCC cells

**Fig. 4:**
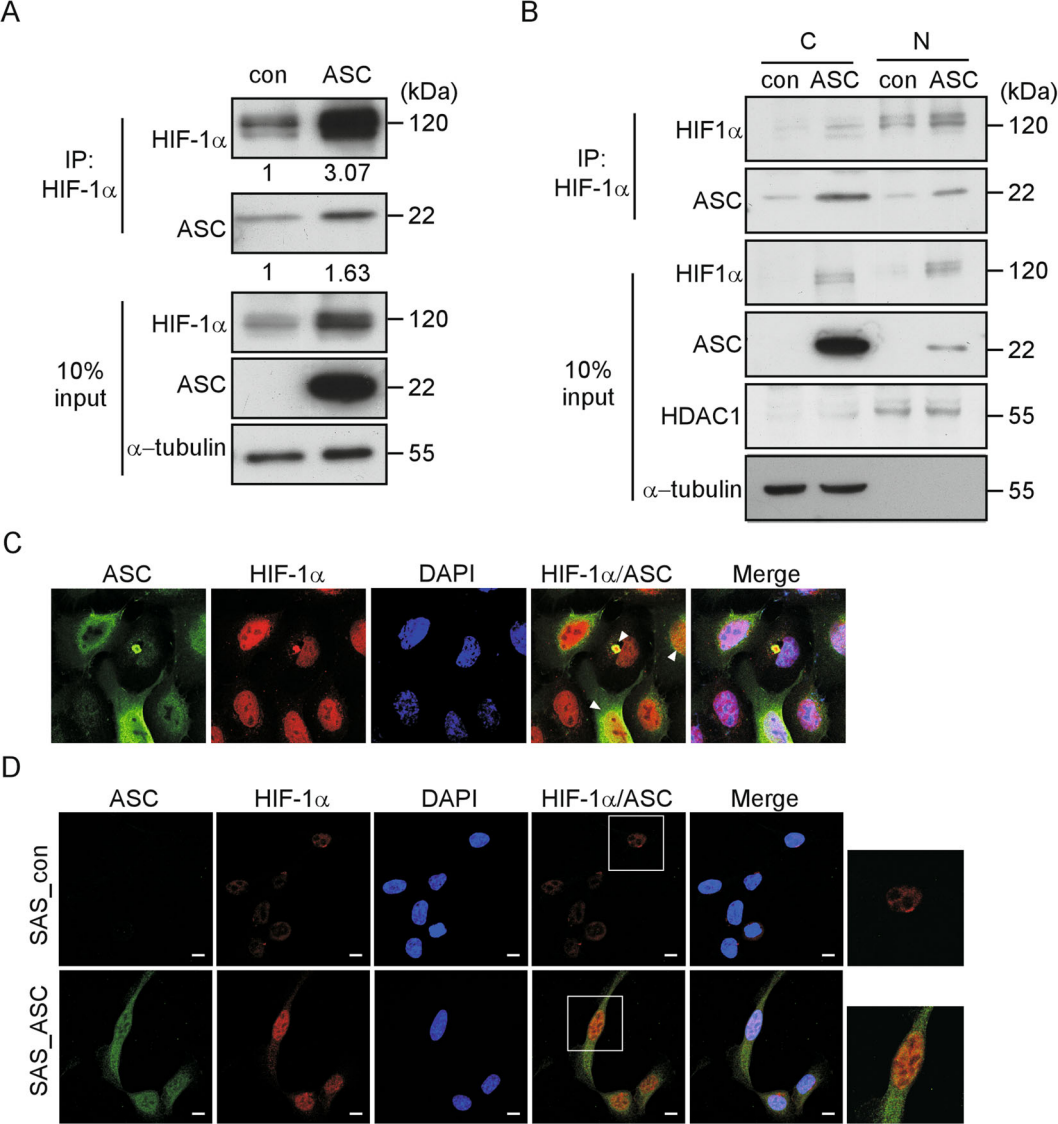
ASC and HIF-1α are colocalized in the cytoplasm and nucleus of SAS_ASC cells. **a** ASC interacts with HIF-1α in SAS_ASC cells. Total cell extracts of SAS_con and SAS_ASC cells were immunoprecipitated with anti-HIF-1α, and the immunoprecipitate was subjected to Western blotting with anti-HIF-1α and anti-ASC. **b** Extracts from cytoplasmic and nuclear fractions of SAS_con and SAS_ASC cells were immunoprecipitated with anti-HIF-1α and western blotting with anti-ASC. C cytoplasm, N nucleus. HDAC1 was used as a nucleus-specific marker. **c** SAS cells were transiently transfected with pLKO.neo.*asc* for 24 h. Immunofluorescence analysis was performed using antibodies specific to ASC (green) and HIF-1α (red), followed by confocal fluorescence microscopy. **d** SAS_con (upper) and SAS_ASC (lower) cells were subjected to immunofluorescence analysis as described in **c**. Scale bar, 10 μm.

As presented above, we found that ASC can stabilize HIF-1α in OSCC cells cultured under normoxia. To elucidate whether the stabilization of HIF-1α involved its physical interaction with ASC in SAS_ASC cells, we used co-immunoprecipitation. As shown in Fig. 4a, ASC clearly co-immunoprecipitated with HIF-1α in total cell extracts of SAS_ASC cells but not in those of SAS_con cells. These data strongly suggest that ASC can form a complex with HIF-1α under the ASC-overexpressed condition. We next questioned whether the ASC-HIF-1α complex could translocate into the nucleus to regulate the downstream cell-motion-associated genes. As shown in Fig. 4b, the amount of HIF-1α was elevated in nuclear fractions of SAS_ASC cells compared to control cells, and the interaction between ASC and HIF-1α was found in both nuclear and cytoplasmic fractions of the former cells. Together, these results indicate that ASC protects HIF-1α from degradation by interacting with it in both the cytoplasm and the nucleus under normoxia.

We also used immunofluorescence to test the localizations of ASC and HIF-1α in OSCC cells. As shown in Fig. 4c, when SAS cells were transiently transfected with an *ASC* overexpression plasmid, we found that ASC and HIF-1α colocalized in the cytoplasm and the nucleus. Similar results were obtained in SAS_ASC cells but not in SAS_con cells (Fig. 4d).

### Repression of HIF-1α inhibits the migration of OSCC cells

**Fig. 5:**
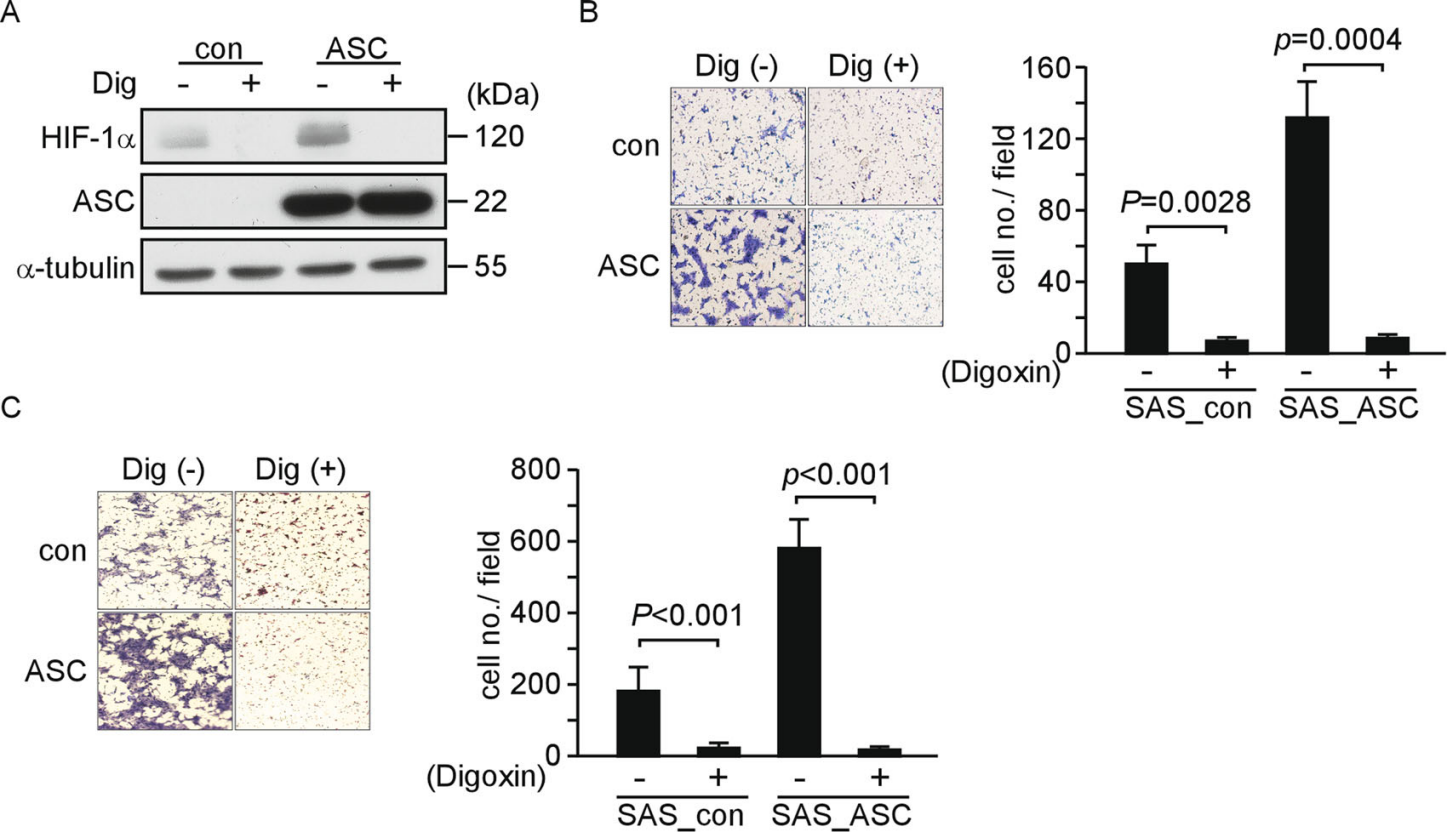
SAS_ASC cell mobility is inhibited by digoxin. **a** HIF-1α was reduced in SAS_ASC cells treated with digoxin. **b** The cell migration ability was reduced in SAS_ASC cells (14-fold decreased, *p* = 0.0028), and in SAS_con cells (five-fold decreased, *p* = 0.0004) (right) after treating with digoxin. **c** Cell invasion was assessed using a strategy similar to that presented in **b**. The cell invasion ability was reduced in both SAS_ASC cells (31.9-fold decreased, *p* < 0.001) and SAS_con cells (6.2-fold decreased, *p* < 0.001) after treating with digoxin.

Inflammasome activity can be inhibited by small molecules, although they tend to be specific to pattern-recognition molecules, such as NLRP3^26^, rather than targeting ASC. As hypoxia is a major hallmark of cancer, various drugs have been developed to inhibit HIF-1α^27^. Digoxin, which has been used in the clinic to treat cardiovascular disease, inhibits HIF-1α synthesis and has been found to reduce tumorigenesis in a variety of cancer cell lines^28–31^. Here, we found that 10 μM of digoxin reduced the protein expression of HIF-1α in SAS_ASC than in SAS_con cells (Fig. 5a). Digoxin treatment reduced the migration ability of SAS_con and SAS_ASC cells by 5- and 14-fold, respectively (Fig. 5b). Similar results were also obtained in our assay of invasion, which decreased by 6.2- and 31.9-fold in SAS_con and SAS_ASC cells, respectively, following digoxin treatment (Fig. 5c).

## Discussion

ASC is well known to mediate inflammasome activation by binding pattern-recognition receptors^32^, but little is known regarding the involvement of ASC in cancer progression^19,33^. We previously reported that overexpression of ASC was correlated with lymph node metastasis in OSCC^6^. Here, we further show that the ASC-HIF-1α regulatory pathway is involved in the lymph node metastasis of OSCC. Ours is the first study to demonstrate an association between ASC and HIF-1α.

GO was used to identify relevant cell-motion-associated genes, and 14 representative genes were found to be overexpressed in SAS_ASC cells and tumor tissues of the OSCC-Taiwan and TCGA datasets. All 14 genes contained potential HREs in their promoter regions; of them, *COL18A1*, *RRAS2*, *CORO1A*, *F2RL1*, and *PTP4A1* had not previously been suggested as being regulatory targets of HIF-1α (Supplementary Table 3). Our prior work showed that *VEGFA, IL6*, and *PDGFB* are overexpressed in OSCC^21^, while other studies found that *THBS1*^34^, *PDGFA*^35^, and *SPHK1*^36^ are upregulated and able to promote migration and invasion activities in OSCC. These six genes were previously reported as being regulated by HIF-1α in OSCC. Moreover, *ICAM1* was reported to be regulated by *IL6* through the Syk/JNK/AP-1 signaling pathway in OSCC.^37^ Here, we found that ICAM1 was upregulated at the protein and gene levels in SAS_ASC cells, suggesting that *ICAM1* may be also regulated by the ASC-HIF-1α pathway. *IRS-1* can be phosphorylated by insulin receptor tyrosine kinase to activate downstream genes, such as *VEGF*. Oxygen tension was found to activate caspase 3 and degrade the IRS-1 protein, but little was known of the relationship between *IRS-1* and HIF-1α^38^. Our present data suggest that *IRS-1* is activated through the ASC-HIF-1α pathway. *CXCL16* was known to be regulated by HIF-1α^39^, but this is the first report to suggest that *CXCL16* may regulate OSCC progression. Of the 14 selected genes, although the gene expression levels of *IL6*, *RRAS2*, *PDGFB*, *SPHK1*, and *VEGF* were upregulated in our cell model system and clinical data, they showed no significant correlation with *ASC*. This may reflect the action of other regulatory signaling pathways or mechanisms that are able to overcome the effects of ASC-HIF-1α signaling^26,28–30^.

**Fig. 6:**
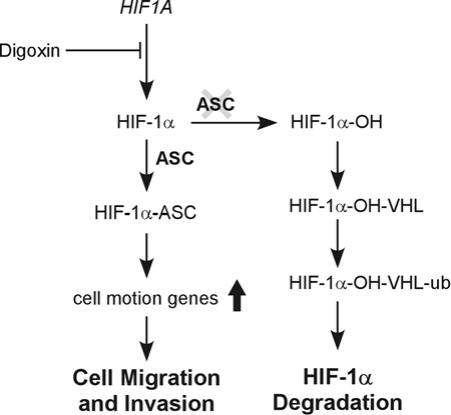
Schematic model of the ASC-HIF-1α regulatory pathway in OSCC. In the canonical HIF-1α pathways, HIF-1α is degraded under normoxia (right panel). However, in the presence of ASC, HIF-1α can be stabilized and then activate its downstream cell motion-associated genes to promote cell migration and invasion (left panel).

In the canonical HIF-1α pathway, HIF-1α is degraded by interacting with VHL protein. Recently, PIN, Erb4, and the long non-coding RNA, *LncHIFCAR*, have been reported to enhance cancer development by interacting with and stabilizing HIF-1α^40–42^. Thus, the activation of HIF-1α under low oxygen tension may be stabilized by its interaction with other molecules. Here, we found that ASC bound to HIF-1α in both the cytoplasm and the nucleus, and subsequently activated downstream genes. We speculate that the binding of ASC to HIF-1α blocks the VHL-interaction site and/or changes the conformation of HIF-1α to reduce the hydroxylation of Pro_402_ and Pro_564_ on its ODD domain^43^. The stabilized HIF-1α then interacts with HIF-1β and other transcription factors to activate the downstream genes. Thus, we propose that HIF-1α is induced not only under a low oxygen tension environment, but also by interacting with ASC under normoxia (Fig. 6).

Under physiological conditions, the tumor microenvironment is hypoxic; this is especially true of the central region of the tumor mass, where HIF-1α is overexpressed^44,45^. At the tumor margin, blood vessels grow to provide sufficient nutrients and oxygen, allowing for normoxia and routine ubiquitin-mediated degradation of HIF-1α. Here, we show that HIF-1α can also be stabilized under normoxia through interacting with ASC in OSCC. This phenomenon was similar to the effect seen in SAS_con cells treated with CoCl_2_ under normoxia. As various cell-motion-associated genes are upregulated by HIF-1α in OSCC and we previously showed that overexpression of ASC promotes lymph node metastasis in OSCC, we speculate that overexpression of ASC in tumor cells of the tumor margin could stabilize HIF-1α activity to sustain the expression of cell migration- or invasion-related genes, enabling the tumor cells to migrate away from the primary site. Through ASC-HIF-1α pathway, we suggest that we could observe lymph node metastasis was increased in mouse model and OSCC patients^6^.

Of the 195 ASC-HIF-1α-regulated cell-motion-associated genes identified herein, 140 were upregulated by more than 1.5-fold in OSCC samples of the TCGA dataset. Among these 140 genes, the overexpression of 16 was associated with poor overall survival in OSCC (data not shown) of the TCGA dataset. These results indicate that ASC is not only involved in inflammasome activation, but also plays a crucial role in tumorigenesis. Thus, our findings identify a novel ASC-HIF-1α pathway that may contribute to OSCC metastasis.

Hypoxia is a hallmark of tumor progression, and many reagents and drugs have been developed to inhibit the effects of hypoxia^27^. Digoxin is a native compound that has been widely used to treat heart failure. Recently, digoxin has also been used to treat tumor progression through the inhibition of HIF-1α synthesis. However, although it acts against many types of cancer cells, it may carry some risks for cancer patients^46^. Although we applied digoxin to decrease the migration and invasion of OSCC cells, little is known regarding the potential usage and toxicity of digoxin for OSCC patients.

In sum, we herein report for the first time that the inflammasome adaptor protein, ASC, can interact with HIF-1α and promote migration and invasion in OSCC cell lines. This is the first study to elucidate the mechanism through which ASC induces cell mobility in OSCC. Based on our present findings, we propose that the ASC-HIF-1α regulatory pathway may offer new a direction for the development of strategies aimed at treating OSCC.

## Materials and methods

### Patient information from the OSCC-TCGA and OSCC-Taiwan databases

To verify the ASC-regulated cell-motion–associated genes were upregulated in the OSCC-TCGA dataset, we selected anatomic neoplasm subdivisions of TCGA HNSC using the following anatomic sites: oral cavity, hard palate, floor of mouth, oral tongue, alveolar ridge, buccal mucosa, and lip. We applied clinical information and gene expression quantification (FPKM) data for survival analysis using TCGABiolinks (version 2.11.1). To perform the Kaplan–Meier analysis, we classified the expression of each gene as being above or below the median FPKM and used the TCGAanalyze_survival function of TCGABiolinks to compute univariate curves for survival. The RNA-seq dataset of OSCC-Taiwan was obtained from a previous study^19^.

### Cell culture

The oral cancer cell line SAS was authenticated by STR and tested for mycoplasma contamination. SAS_con and SAS_ASC cell lines were established by transfection with pLKO.AS2.neo and pLKO.AS2.neo.ASC vectors, respectively. The two cell lines were maintained in DMEM containing 10% FBS, G418 (200 μg/ml), and puromycin (2 μg/ml) (Thermo Fisher Scientific, CA, USA), as previously reported^6^.

### Identification of upstream transcription factors

Ingenuity Upstream Regulator Analysis in IPA software (QIAGEN, CA, USA) was used to predict the upstream regulator of the high throughput dataset^47^. Briefly, 3513 differential expressed genes (1806 downregulated and 1707 upregulated) between SAS_con and SAS_ASC cells were selected and subjected to the software analysis. We identified 508 upstream regulators in which 66 are known transcription factors, and 27 of the 66 transcription factors were found activated (*p* < 0.05, z-score>2) in both IPA prediction and RNA-seq data (Supplementary Table 4).

### Western blot and antibodies

Western blot analysis was followed the protocol reported before^48^. Briefly, SAS_con and SAS_ASC cells were lysed in RIPA buffer. Fifty microgram of protein was subjected to the following analyses and incubated with primary antibodies for individual proteins at 4 °C overnight and then incubated with HRP-conjugated rabbit/mouse anti-IgG (Thermo Fisher Scientific, CA, USA) for 1 h at room temperature. Proteins were detected using an ECL system (Thermo Fisher Scientific, CA, USA) and X-ray film. The primary antibodies included anti-ASC antibody (D086-3) from MBL International Corporation (MA, USA); anti-HIF-1α (610958) and anti-VHL (564183) from BD Bioscience (CA, USA); anti-Ubiquitin (ab7780) and anti-HIF-1β (ab2771) from Abcam (Cambridge, UK); anti-HIF-1α-OH (D43B5) Cell Signaling (MA, USA); anti-HIF-1α (sc-13515), anti-PHD (sc-271835), anti-HDAC1 (sc-81598), anti-PDGFA (sc-9974), anti-CORO1A (sc-100925), anti-ICAM1 (sc-8439), anti-THBS1 (sc-59887), and anti-α-Tubulin (sc-32293) from Santa Cruz Biotechnology (TX, USA).

### Statistics

The data obtained from the qRT-PCR, migration, invasion, and RNA-seq analyses of tumor and adjacent normal tissues of OSCC-Taiwan samples were all analyzed by the Student’s *t*-test. The significance of expressional differences between tumor tissues and normal control tissues of TCGA samples was assayed by the Mann–Whitney U test. Results of independent western blots of individual proteins were quantified by software Image J, and the data shown as mean ± SD. A *p* value less than 0.05 was considered statistically significant.

## Supplementary information

Supplementary Information

Supplementary Figure legends

Supplementary Tables

Supplementary Figure 1

Supplementary Figure 2

Supplementary Figure 3

Supplementary Figure 4
