# Supplementary material for: ASC modulates HIF-1α stability and induces cell mobility in OSCC
Source: Cell Death Dis. 2020 Sep 3;11(9):721. doi: 10.1038/s41419-020-02927-7 (PMC7471912; doi:10.1038/s41419-020-02927-7)
Supplement: Supplementary file 1 — Supplementary Information [file 41419_2020_2927_MOESM1_ESM.docx]

Supplementary Information for

**ASC modulates HIF-1α stability and induces cell mobility in OSCC**

Chi-Sheng Wu^1,2*^, Ian Yi-Feng Chang ^1^, Jui-lung Hung^1^, Wei-Chao Liao^1,3^, Yi-Ru Lai^4^, Kai-Ping Chang^1,2,6^, Hsin-Pai Li^1,4,5^, Yu-Sun Chang^1*^

*Corresponding authors.

Tel.: +886 3 2118800x5107; Fax: +886 3 2118683 (C.-S. Wu).

E-mail addresses: [wcs573@mail.cgu.edu.tw](mailto:wcs573@mail.cgu.edu.tw) (C.-S. Wu).

Tel.: +886 3 2118800x5131; Fax: +886 3 2118683 (Y.-S. Chang).

E-mail addresses: [ysc@mail.cgu.edu.tw](mailto:ysc@mail.cgu.edu.tw) (Y.-S. Chang).

**Materials and Methods**

**RNA extraction, NGS and qRT-PCR analysis**

Total RNA was prepared from OSCC cells using the TRIZOL reagent (Invitrogen). Total RNA isolated from 6×10^5^ SAS_con and SAS_ASC cells was subject to library preparation using a TruSeq RNA Library Prep Kit v2 (Illumina) and sequenced on a NextSeq500 (Illumina) as described by the manufacturer. The differentially expressed genes were identified with the Partek data analysis software (Partek Inc.). Raw data were deposited into Gene Expression Omnibus under accession number GSE150920. The cDNA was generated with an MMLV reverse transcriptase system (Invitrogen) as described by the manufacturer. Quantitative real time PCR (qRT-PCR) was performed using a Light-cycler (Roche). The expression level of TATA-binding protein (TBP) was monitored as an internal control. The utilized primers are listed in Supplementary Table 5. All qRT-PCR analyses were performed in duplicate for three independent tests.

**ASC knockdown test**

ASC knockdown analysis was performed as previous study[^6^](#_ENREF_6). Briefly, 2×10^5^ SAS_con and SAS_ASC cells were seeded to 6-well culture plates. Cells were transient transfected with scrambled or ASC specific siRNAs (Dharmacon, CO, USA) for ASC knockdown studies. After 48 hours, cells were harvested and performed western blot analysis of ASC using anti-ASC antibody (MBL) and HIF-1α using anti- HIF-1α antibody (BD).

**CoCl_2_ induced hypoxia in OSCC cells**

To test if HIF-1α was induced by CoCl_2_ in OSCC cells, 3.5×10^5^ SAS_con and SAS_ASC cells were cultured in DMEM with 10% FBS and treated with 100 μM of CoCl_2_ for 20 hours. Cells were harvested and examined the protein level of ASC and HIF-1α by western blot analysis using specific antibodies as described above.

**Co-immunoprecipitation**

For co-immunoprecipitation, 20 μl of Protein-G-Sepharose was washed three times with homo-buffer (10 mM Tris-HCl, 1 mM EDTA, 1 mM EGTA, 50 mM NaCl, 50 mM NaF, 20 mM Na_4_P_2_O_7_, and 1 mM Na_3_VO_4_, PH 7.4). One mg of total protein extract was pre-cleaned by incubation with the prepared Protein-G-Sepharose for 1 hours at 4℃. The pre-cleaned protein extract was transferred to a new Eppendorf tube and incubated overnight at 4℃ with 1 μg of anti-HIF-1α (BD) for immunoprecipitation. On the following day, Protein-G-Sepharose was pre-cleaned with washing buffer (20 mM Tris, 0.5 mM DTT, 0.5M NaCl) and incubated with the IP product for 1 hours at 4℃. The Protein-G-Sepharose-conjugated IP product was collected by centrifugation at 6000 rpm for 3 minutes at 4℃ and washed twice with washing buffer (20 mM Tris, 0.5 mM DTT). The collected samples were mixed with 20 μl of sample buffer and subjected to Western blot analysis. For extraction of cytoplasmic and nuclear proteins, 2×10^7^ SAS_con and SAS_ASC cells were washed three times with 1×PBS and processed with a Protein Extraction kit (BRARZ106, TOOLS). Briefly, cell pellets were mixed with 200 μl of Cytoplasmic Extraction Reagent (CER) containing 1 mM of PMSF, vortexed for 15 seconds, and incubated for 20 minutes at room temperature. The samples were centrifuged at 12,000 g for 5 minutes at 4℃, and the supernatants were collected as the cytoplasmic protein fraction. The pellet was incubated with 500 μl of Nuclear Protein Reagent (NER), vortexed the solution well 10 to 20 seconds each time, at intervals of 5 minutes for 6 times. The samples were centrifuged at 12,000 g for 10 minutes, and the supernatants were collected as the nuclear protein fraction. The obtained cytoplasmic and nuclear proteins could be directly used for IP.

**Immunofluorescence Microscopy**

3.5×10^5^ SAS_con and SAS_ASC cells were grown on coverslips for 16 hours. The cells were fixed with 3.7% formaldehyde, permeated and blocked with 0.1% sponin containing 1% BSA for 20 minutes. The coverslips were incubated with anti-ASC (MBL, MA, USA) and anti-HIF-1α (Santa Cruz, TX, USA) antibodies for 2 hours, and then with fluorophore-conjugated secondary antibodies (Alexa Fluor Goat anti-mouse/rabbit, Thermo Fisher Scientific, CA, USA) for 1 hour at room temperature. Nuclei were stained with 4-6-diamidino-2-phenylindole (DAPI, Sigma-Aldrich). The coverslips were mounted with VECTASHEILD reagent (Vector Laboratories Inc., CA, USA) and visualized by confocal microscopy ZEISS LSM510 META laser scanning microscope (Carl Zeiss, Germany) with 63×1.32 NA oil immersion objective.

**Trans-well migration assay**

The 24-well trans-well migration assay was performed according to the manufacturer’s protocol (Corning)[^6^](#_ENREF_6). Briefly, 1×10^5^ SAS_con and SAS_ASC cells were washed twice with serum-free DMEM, re-suspended in 100 μl serum-free medium, and loaded into the upper chamber. The lower chamber was loaded with 750 μl DMEM containing 10% FBS. The plates were incubated under 5% CO_2_ at 37℃ for 16 hours, and the cells that migrated through and attached on the membrane were fixed and stained with 0.25% crystal violet, 10% formaldehyde, and 80% methanol for 15-20 minutes, and then washed with ddH_2_O. Cells were counted by averaging the cell number obtained from 10 random microscopic fields under 100 × magnification. The test was performed three independent times.

**Cell invasion assay**

Cell invasion was assessed using a Corning Invasion kit (#354480, Corning) as described by the manufacturer. Briefly, the ECM layer in the chamber was re-hydrated with serum-free DMEM for 2 hours at 37℃. The insert was loaded with 1×10^5^ SAS_con or SAS_ASC cells in 350 μl serum-free DMEM, while the lower chamber was loaded with 750 μl 10% DMEM. The chambers were incubated in 5% CO_2_ at 37℃ for 24 hours, and the non-invaded cells were gently removed with a cotton swab. Staining and counting of the invaded cells was performed as described above for the migration assay. The results were obtained from three independent experiments.

**Digoxin treatment**

For the HIF-1α inhibition test, 6×10^5^ SAS_con and SAS_ASC cells were treated with 10 μM of digoxin (AbMole Bioscience, NC, USA). After 24 hours, the cells were harvested and subjected to trans-well migration and invasion assays.
