# Supplementary material for: ASC modulates HIF-1α stability and induces cell mobility in OSCC
Source: Cell Death Dis. 2020 Sep 3;11(9):721. doi: 10.1038/s41419-020-02927-7 (PMC7471912; doi:10.1038/s41419-020-02927-7)
Supplement: Supplementary file 2 — Supplementary Figure legends [file 41419_2020_2927_MOESM2_ESM.docx]

**Supplementary figure legends**

**Supplementary Figure 1. Gene expression levels of ASC, as analyzed by next-generation sequencing (NGS).** NGS was used to assess the gene expression levels of ASC in (**A**) OSCC-Taiwan (paired normal/tumor tissues, n=39) and (**B**) the OSCC-TCGA dataset (n=30 for normal control, n=308 for OSCC).

**Supplementary Figure 2.**  **Kaplan-Meier survival analysis of patients exhibiting up-regulation of *PDGFA*, *RRAS2*, and *VEGFA* in the TCGA-OSCC dataset.** The y-axis indicates the OS probability, while the x-axis indicates the live days of each individual. The red and blue lines indicate the patients with high- and low-level expression of *PDGFA*(left), *RRAS1*(middle), and *VEGFA*(right), as determined from the RNA-seq data. The up-regulations of these genes were correlated with poor OS in OSCC.

**Supplementary Figure 3. Protein expression levels of the putative ASC-regulated genes in SAS_ASC cells. A**. PDGFA, CORO1A, ICAM1, and THBS1 proteins levels were up-regulated In SAS_ASC cells compared to control cells. **B**. PDGFA, CORO1A, ICAM1, and THBS1 proteins were down-regulated after SAS_ASC cells were treated with siRNA specific to ASC. **C**. Protein levels of targets detected in our previous proteomic analysis of 39 normal/tumor paired OSCC samples.

**Supplementary Figure 4. Expression of genes and proteins in the HIF-1α signaling. A** to **G.** HIF-1α protein expression is stabilized in the SAS_ASC cell line, presenting of the other two of the three independent western blot experiments shown in Figure 3B to 3H. **H**. Gene expression levels of *vhl*, *hif1b*, and *phd2*, as analyzed by qRT-PCR. The y-axis indicates the fold change in SAS_ASC cells relative to SAS_con cells. **I.** The other independent co-IP test of in Figure 3I.
