# Supplementary material for: ASC modulates HIF-1α stability and induces cell mobility in OSCC
Source: Cell Death Dis. 2020 Sep 3;11(9):721. doi: 10.1038/s41419-020-02927-7 (PMC7471912; doi:10.1038/s41419-020-02927-7)
Supplement: Supplementary file 3 — Supplementary Tables [file 41419_2020_2927_MOESM3_ESM.docx]

| **Supplementary Table 1. Gene Ontology of 195 ASC induced cell-motion-associated genes** | | | | | | |  |  |
| --- | --- | --- | --- | --- | --- | --- | --- | --- |
| **GO** |  | **Fold Enrichment** |  | ***p*-Value** |  | **Benjamini** |  | **FDR** |
| positive regulation of cell motion |  | 11.31 |  | 4.69E-11 |  | 8.83E-08 |  | 8.01E-08 |
| positive regulation of cell migration |  | 11.63 |  | 1.78E-10 |  | 1.67E-07 |  | 3.03E-07 |
| positive regulation of locomotion |  | 10.56 |  | 6.11E-10 |  | 3.83E-07 |  | 1.04E-06 |
| regulation of cell motion |  | 6.89 |  | 9.10E-10 |  | 4.28E-07 |  | 1.55E-06 |
| regulation of cell migration |  | 7.44 |  | 1.01E-09 |  | 3.80E-07 |  | 1.72E-06 |
| regulation of cell proliferation |  | 3.29 |  | 1.15E-09 |  | 3.61E-07 |  | 1.97E-06 |
| regulation of locomotion |  | 6.55 |  | 6.50E-09 |  | 1.75E-06 |  | 1.11E-05 |
| cell adhesion |  | 3.27 |  | 1.53E-08 |  | 3.59E-06 |  | 2.61E-05 |
| biological adhesion |  | 3.27 |  | 1.58E-08 |  | 3.30E-06 |  | 2.69E-05 |
| response to organic substance |  | 3.18 |  | 2.97E-08 |  | 5.59E-06 |  | 5.07E-05 |
| wound healing |  | 6.19 |  | 4.36E-08 |  | 7.46E-06 |  | 7.44E-05 |
| response to wounding |  | 3.63 |  | 4.45E-08 |  | 6.98E-06 |  | 7.60E-05 |
| regulation of apoptosis |  | 2.94 |  | 9.58E-08 |  | 1.39E-05 |  | 1.64E-04 |
| regulation of programmed cell death |  | 2.91 |  | 1.20E-07 |  | 1.61E-05 |  | 2.04E-04 |
| regulation of cell death |  | 2.9 |  | 1.30E-07 |  | 1.63E-05 |  | 2.22E-04 |
| positive regulation of cell proliferation |  | 3.93 |  | 1.76E-07 |  | 2.07E-05 |  | 3.01E-04 |
| response to hormone stimulus |  | 4.03 |  | 5.19E-07 |  | 5.74E-05 |  | 8.85E-04 |
| response to endogenous stimulus |  | 3.83 |  | 5.40E-07 |  | 5.65E-05 |  | 9.22E-04 |
| positive regulation of apoptosis |  | 3.61 |  | 1.38E-06 |  | 1.36E-04 |  | 2.35E-03 |
| positive regulation of programmed cell death |  | 3.59 |  | 1.53E-06 |  | 1.44E-04 |  | 2.61E-03 |
| (https://david.ncifcrf.gov/) |  |  |  |  |  |  |  |  |

**Supplementary Table 2. Pathways of 195 ASC induced cell motion associated genes**

| **Pathway** | ***p*-Value** | **Benjamini** |
| --- | --- | --- |
| Pathways in cancer | 1.00E-05 | 1.10E-03 |
| Focal adhesion | 1.40E-05 | 7.40E-04 |
| ECM-receptor interaction | 5.30E-05 | 1.80E-03 |
| Cytokine-cytokine receptor interaction | 3.00E-04 | 7.70E-03 |
| Small cell lung cancer | 3.20E-04 | 6.60E-03 |
| Regulation of actin cytoskeleton | 4.80E-03 | 8.00E-02 |
| Folate biosynthesis | 2.10E-02 | 2.70E-01 |
| Hematopoietic cell lineage | 3.30E-02 | 3.50E-01 |
| NOD-like receptor signaling pathway | 4.07E-02 | 3.79E-01 |
| Chemokine signaling pathway | 4.00E-02 | 3.80E-01 |
| Jak-STAT signaling pathway | 4.20E-02 | 3.60E-01 |
| MAPK signaling pathway | 5.00E-02 | 3.80E-01 |
| p53 signaling pathway | 5.30E-02 | 3.70E-01 |
| (https://david.ncifcrf.gov/) |  |  |

**Supplementary Table 3. The correlation of cell motion genes with *ASC* and overall survival in OSCC-Taiwan and -TCGA datasets**

| **Genes** | **Chr.** | Correlate with  *ASC* in OSCC-Taiwan | | OS in TCGA |
| --- | --- | --- | --- | --- |
|  |  | ***r*** | ***p*-Value** | ***p*-Value** |
| *COL18A1^*^* | 21 | 0.41 | 0.01 | 0.42 |
| *IL6* | 7 | 0.27 | 0.11 | 0.95 |
| *ICAM1* | 19 | 0.39 | 0.01 | 0.49 |
| *PDGFA* | 7 | 0.41 | 0.01 | 0.01 |
| *RRAS2^*^* | 11 | 0.19 | 0.22 | 0.03 |
| *CORO1A^*^* | 16 | 0.41 | 0.01 | 0.09 |
| *THBS1* | 15 | 0.33 | 0.03 | 0.47 |
| *F2RL1^*^* | 5 | 0.43 | 0.00 | 0.28 |
| *IRS1* | 2 | 0.30 | 0.05 | 0.85 |
| *CXCL16* | 17 | 0.38 | 0.01 | 0.43 |
| *PDGFB* | 22 | 0.11 | 0.48 | 0.53 |
| *VEGFA* | 6 | 0.07 | 0.68 | 0.02 |
| *SPHK1* | 17 | 0.27 | 0.08 | 0.12 |
| *PTP4A1^*^* | 6 | 0.34 | 0.03 | 0.15 |

^*^: the gene was predicted regulated by HIF-1α in this study

**Supplementary Table 4. Activated upstream transcription factors in SAS_ASC cell line** **(z-score>2)**.

| **Upstream Regulator** | **Expr Fold Change** | **Activation z-score** | ***p*-Value** |
| --- | --- | --- | --- |
| HIF1A | 1.228 | 3.832 | 3.63E-14 |
| STAT3 | 1.311 | 3.09 | 4.80E-12 |
| IRF1 | 1.952 | 3.472 | 1.52E-10 |
| STAT1 | 1.187 | 5.125 | 2.36E-08 |
| IRF3 | 1.069 | 3.193 | 3.29E-08 |
| STAT4 | 1.035 | 2.484 | 8.71E-08 |
| IRF7 | 2.328 | 4.081 | 1.37E-07 |
| RELA | 1.13 | 2.353 | 2.52E-07 |
| PML | 1.195 | 2.415 | 2.67E-07 |
| EPAS1 | 1.118 | 2.268 | 3.33E-07 |
| KLF4 | 1.746 | 3.008 | 7.97E-07 |
| CEBPB | 1.36 | 2.039 | 8.31E-07 |
| STAT2 | 1.11 | 2.623 | 1.68E-06 |
| ETS1 | 2.054 | 2.36 | 1.95E-06 |
| DDIT3 | 1.211 | 2.815 | 3.01E-06 |
| SMAD3 | 1.075 | 2.564 | 2.15E-05 |
| KLF6 | 1.717 | 2.27 | 2.18E-05 |
| ATF2 | 1.009 | 2.547 | 4.53E-05 |
| EP300 | 1.02 | 2.022 | 5.62E-05 |
| FOXL2 | 1.019 | 3.098 | 2.83E-04 |
| KLF5 | 1.115 | 2.028 | 5.68E-04 |
| E2F3 | 1.05 | 3.422 | 2.13E-03 |
| MTPN | 1.026 | 2.147 | 3.20E-03 |
| HDAC7 | 1.075 | 2.457 | 3.68E-03 |
| NFKBIB | 1.15 | 2.106 | 9.75E-03 |
| IFI16 | 1.003 | 3.204 | 1.30E-02 |
| CEBPD | 1.163 | 2.314 | 1.46E-02 |

**Supplementary Table 5. Primers used in this study.**

| COL18A1 F  COL18A1 R  ICAM F | CTCGAGGTGGACACCACCCT  CAGCTGGATGGCCACATCGG  CAAGGTGACCGTGAATGT |
| --- | --- |
| ICAM R | TTCCGCTGGCGGTTATAGA |
| IRS1 F | TACAGGGTGGGCCAAATTA |
| IRS1 R | AAATGTTTTGGTAGCAATACAGAC |
| RRAS2 F | AATGTCAGCCTTTGTTAACC |
| RRAS2 R | AATCCATAAATACAACAGGCA |
| COROA1 F | GGGAGCTGAGGGTCAAC |
| COROA1 R | CATCTCCTCCTCCAGCC |
| THBS1 F | CATGACCCTCGTCACATAG |
| THBS1 R | TTATCATAGATGGGTCCTGAGT |
| F2RL1 F | CCATCCAAGGAACCAGTAGA |
| F2RL1 R | TGCAGAAAACTCATCCACAGA |
| IRS1 F | TACAGGGTGGGCCAAATTA |
| IRS1 R | AAATGTTTTGGTAGCAATACAGAC |
| CXCL16 F | CATGGGTTCAGGAATTGATG |
| CXCL16 R  PDGFA F  PDGFA R | CCTCTGAGGCCTGAGAA  AggTTAgAggAgCATTTgg  ACTggCAATAAAgCACCgTA3 |
| PDGFB F | GCAAGCACCGGAAATTCAA |
| PDGFB R | GGGCAATACAGCAAATACCATA |
| SPHK1 F | GGCTGAGGCTGAAATCT |
| SPHK1 R | GCCCGTCTCCAGACATGA |
| PTP4A1 F | CAGATTGTTGATGACTGGTTAAG |
| PTP4A1 R | CGTATTTCATTCCACCTTCAATTA |
| VEGFA F | GGTATAAGTCCTGGAGCGT |
| VEGFA R | CTGCGGATCTTGTACAAAC |
| TBP F  TPB R | TGCTCACCCCACCAACAATTTAG  CTGGGTTTGATCATTCTGTAGATTAA |
