# Supplementary figures and images for: ASC modulates HIF-1α stability and induces cell mobility in OSCC
Source: Cell Death Dis. 2020 Sep 3;11(9):721. doi: 10.1038/s41419-020-02927-7 (PMC7471912; doi:10.1038/s41419-020-02927-7)

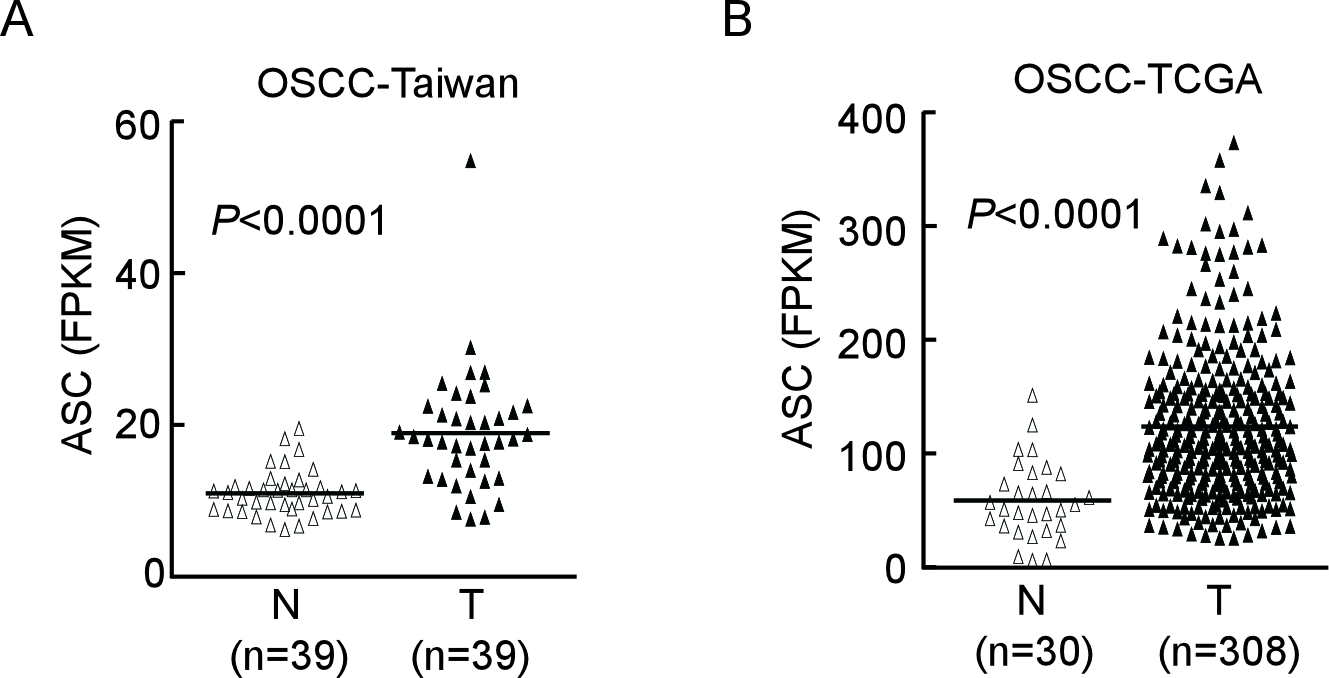

Supplement: Supplementary file 4 — Supplementary Figure 1 [file 41419_2020_2927_MOESM4_ESM.tif]

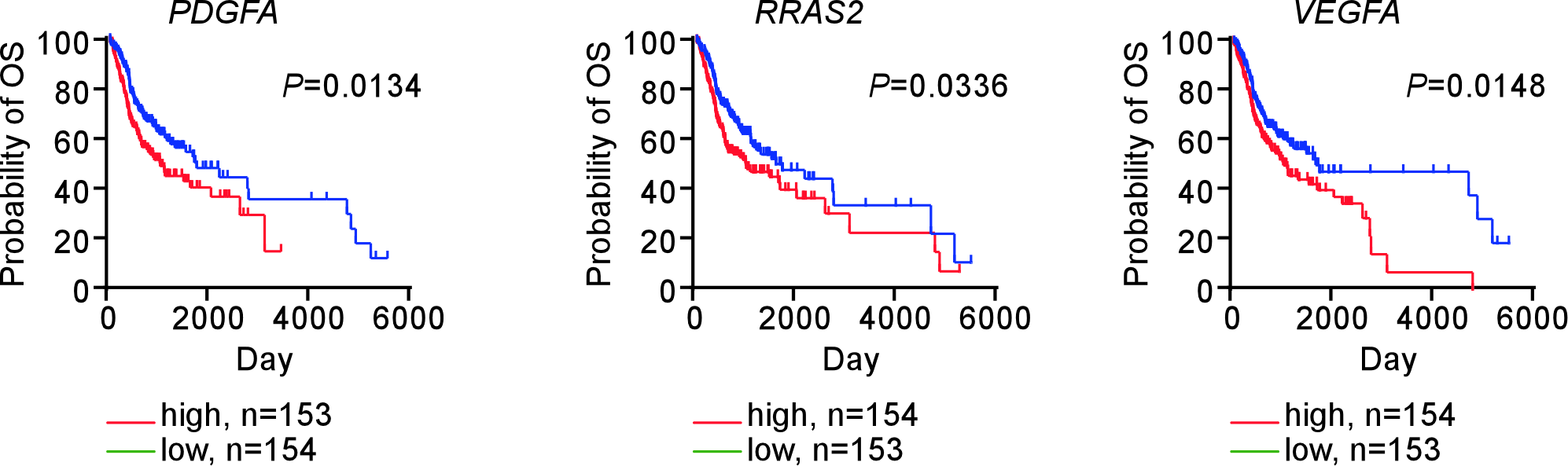

Supplement: Supplementary file 5 — Supplementary Figure 2 [file 41419_2020_2927_MOESM5_ESM.tif]

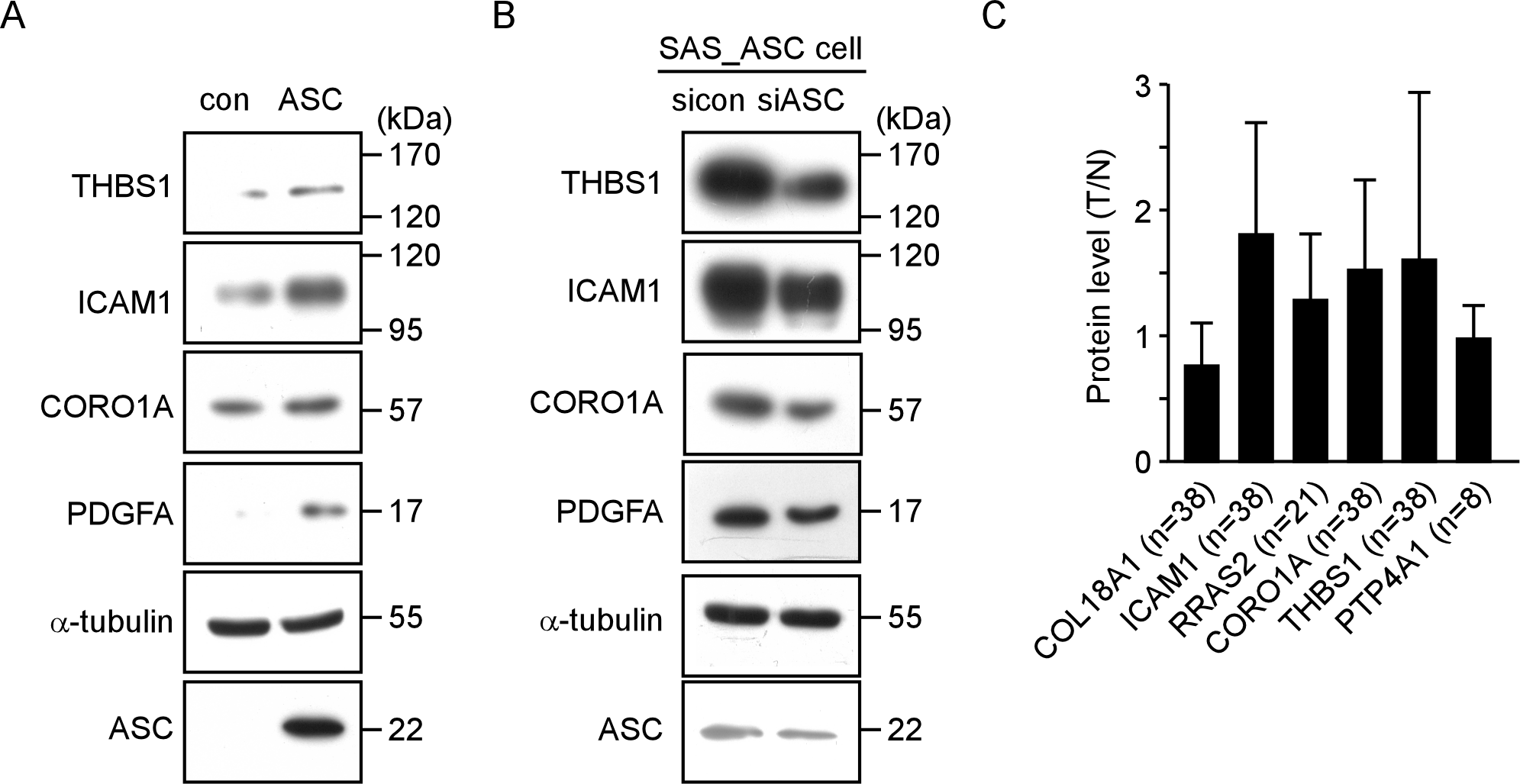

Supplement: Supplementary file 6 — Supplementary Figure 3 [file 41419_2020_2927_MOESM6_ESM.tif]

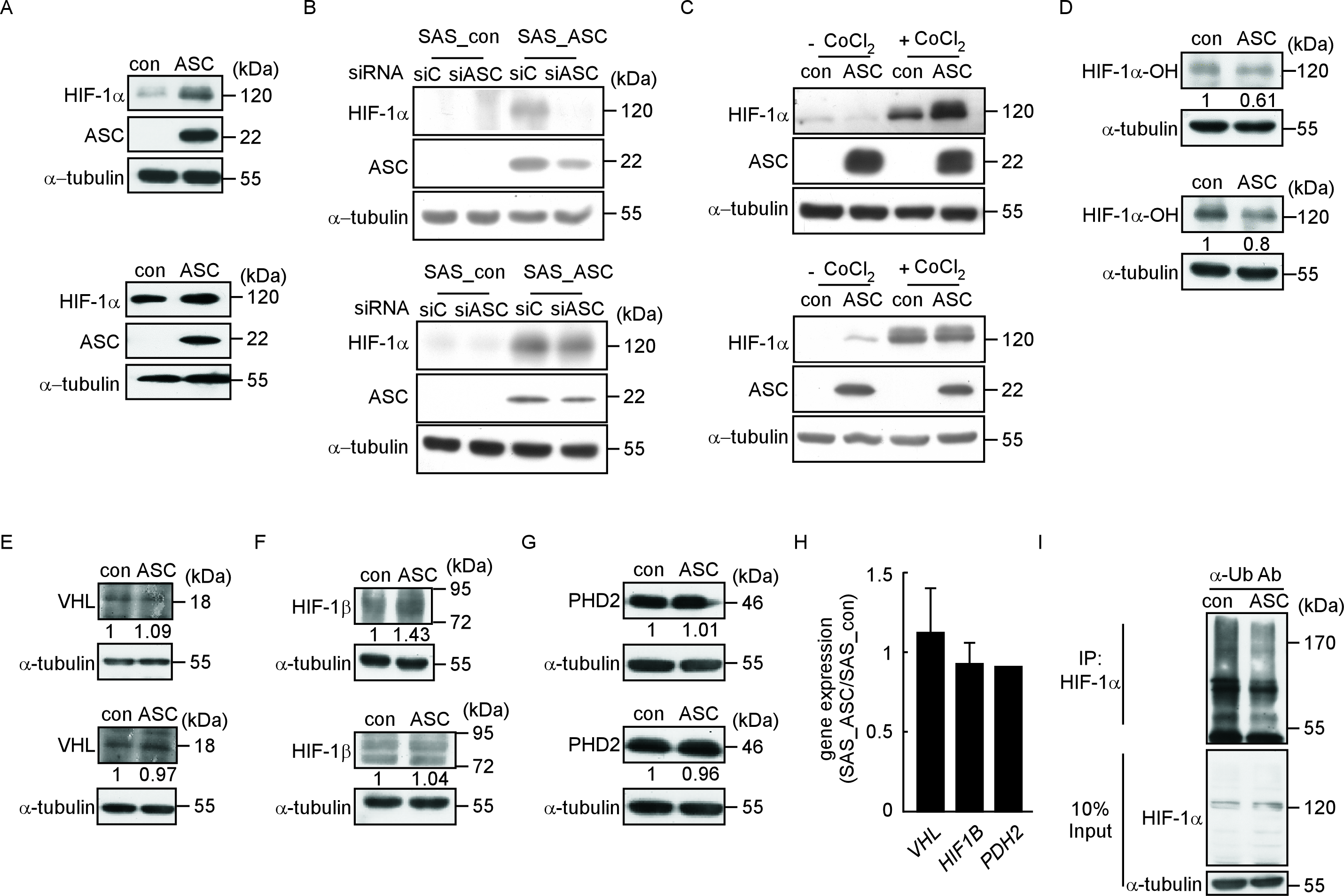

Supplement: Supplementary file 7 — Supplementary Figure 4 [file 41419_2020_2927_MOESM7_ESM.tif]
